# Prevention of *Babesia canis* in dogs: efficacy of a fixed combination of permethrin and fipronil (Effitix®) using an experimental transmission blocking model with infected *Dermacentor reticulatus* ticks

**DOI:** 10.1186/s13071-015-0645-4

**Published:** 2015-01-17

**Authors:** Christelle Navarro, Nadège Reymond, Josephus Fourie, Klaus Hellmann, Stéphane Bonneau

**Affiliations:** Virbac, 13ième rue - LID, 06511 Carros, France; Nadege Savelli EIRL, 2, rue de l’église, 06 270 Villeneuve Loubet, France; ClinVet International, P.O. Box 11186, 9321 Universitas, South Africa; KLIFOVET AG, Geyerspergerstr. 27, 80689 Munich, Germany

**Keywords:** Babesia canis, Dermacentor reticulatus, Ticks, Ectoparasiticides, Fipronil, Permethrin, Transmission blocking

## Abstract

**Background:**

Two experimental studies using a transmission blocking model with *Dermacentor reticulatus* ticks infected with *Babesia canis* were performed to test the ability of Effitix® to prevent the transmission of babesiosis in dogs.

**Methods:**

Four groups of seven dogs (experiment 1) and one group of eight dogs (experiment 2) were treated topically with a novel combination of fipronil and permethrin in a spot-on formulation (Effitix®, Virbac) respectively 28, 21, 14 and 7 days (experiment 1) and 2 days (experiment 2) prior to tick infestation. In each study, a control group of seven dogs (experiment 1) and eight dogs (experiment 2) remained untreated. On day 0, all dogs were infested with adult *D.reticulatus* ticks harboring *B. canis.* An efficacy failure (successfully infected) was regarded as a dog in the treated groups that was tested serologically positive for *B.canis* antibodies, using an indirect fluorescent antibody (IFA) assay and tested positive for *B.canis* by DNA-assay using PCR analysis.

**Results:**

*B.canis* was transmitted by *D.reticulatus* to all untreated dogs (experiment 1) and six untreated dogs out of eight (experiment 2) as confirmed by IFA and PCR assays. The large majority of treated dogs (92.9% in experiment 1 and 100% in experiment 2) remained sero-negative over the challenge period.

**Conclusions:**

The treatment of dogs with Effitix® applied 2 to 28 days prior to infestation with *D. reticulatus* harboring *B.canis*, successfully prevented the transmission of canine babesiosis.

## Background

Canine babesiosis is a tick-borne protozoan disease of worldwide significance. The main clinical signs described in dogs are lethargy, anorexia, hyperthermia (≥39°C) followed by pale mucous membranes, discoloration of the urine and splenomegaly [1]. The most frequent causative agent of canine babesiosis in Central Europe is *Babesia canis*. This parasite is an intraerythrocytic protozoa which activates antibody-mediated cytotoxic destruction of erythrocytes leading to death, in case of massive infection [2]. Historically, *Babesia* parasites in dogs were divided into two groups based on morphological distinction: the larger *B.canis* and the smaller *Babesia gibsoni*. Based on cross-immunity and vector-specificity, *B.canis* has been then reclassified into three subspecies: *B. canis canis*, *B.canis rossi* and *B.canis vogeli*. They are now considered as three separate species: *B.canis*, *B.rossi* and *B.vogeli* [3].

The incidence of clinical babesiosis varies amongst countries and regions in Europe. *B.canis* was historically located in central and eastern regions [3]. In France, the disease is endemic with an overall presence of babesiosis due to *B.canis* with nevertheless local variations. In Spain and Hungary *B.canis* is endemic with several species of piroplasms co-existing in Spain. In Romania, a recent survey has indicated that infection with *B.canis* in dogs is common, and that it is an important pathogen for the local canine population. In Italy, a high seroprevalence of *B.canis* was shown in kenneled dogs. However, the custom of traveling with family pets or hunting dogs on trips to distant regions and returning home has led to an alarming increase in reports of canine vector-borne pathogens in northern and cooler regions of mainland Europe where these diseases were previously unreported. In the United Kingdom, Benelux, Germany, Switzerland and Austria, imported cases or small autochthonous foci of *B.canis* infection have been reported even if there is so far an overall low prevalence of the disease [3-9]. This adds to the relevance of practicing effective tick control on dogs.

Effitix® is a combination of two active ingredients fipronil 6.1% and permethrin 54.5% (w/v) in a solution for topical application (spot-on). Fipronil has well-established insecticidal and acaricidal properties [10]; permethrin brings to the combination its strong repellent effects resulting in absence or reduction of the blood feeding (anti-feeding effect) [11-13].

In the two studies presented below, we tested the ability of Effitix® to prevent transmission of *B.canis* to dogs based on an experimental model recently described in the literature [14-17] and developed to assess the efficacy in preventing the transmission of tick-borne pathogens using *Dermacentor reticulatu s*ticks infected with *B.canis*.

## Methods

### General

The two studies were conducted according to the International Cooperation on Harmonization of Technical Requirements for Registration of Veterinary Medicinal Products Guideline (VICH GL9): Good Clinical Practice [18]and in compliance with local animal welfare legislation. They were performed at the same test facilities in South Africa and followed very similar but complementary protocols. The two protocols were approved by the ClinVet animal ethics committee (references CV12/949 and CV13/091). Both studies investigated the efficacy of Effitix® in reducing the transmission of *B.canis*, from two days (Experiment 2) up to 28 days (Experiment 1) after application.

### Study design, animal selection and treatment

The studies were parallel group designed, randomized, controlled, blinded and experimental efficacy studies. In order to control bias, the products were not administered by an individual involved in performing the post-administration assessments and observations. The dogs were randomly allocated to group according to their body weight within gender at inclusion. All dogs were dewormed and did not harbour any ticks at the initiation of the study. At least during the 12 weeks preceding the treatment, the dogs had not been treated with a long acting topical or systemic acaricide/insecticide. They were sero-negative for *B.canis* prior to initiation of acclimatisation. Animals were treated once at two spots (equal volumes), one between the shoulder blades and the second at the lumbar area directly to the skin. The size of the pipette of the product to apply was chosen according to the body weight of the dog and followed the label recommendations *i.e.* one 1.1 ml pipette of fipronil 6.1% and 54.5% permethrin (w/v) solution for dogs weighing >4-10 kg, one 2.2 ml pipette for dogs weighing >10-20 kg, one 4.4 ml pipette for dogs weighing >20-40 kg.

### Tick challenges

A laboratory-bred *D.reticulatus* tick strain, infected with *B.canis,* was used for the artificial challenges. A sample of 50 ticks was taken from the batch of ticks used for artificial challenges and the infectivity confirmed by PCR analysis. On day 0, each dog was infested with 50 (±4) viable, unfed adult *D.reticulatus* with a balanced sex ratio (50% female:50% male). Ticks were applied directly onto the dog by tapping the vial to dislodge the ticks from the container so that they were placed or spread directly over the dog’s hair coat. Dogs were restrained for 10 minutes and confined in an infestation chamber to enhance tick attachment for approximately four hours. Ticks dislodged during the first 10 minutes were placed back onto the dog.

### Tick counts

Following infestation, *in situ* thumb counts were performed regularly. The number of ticks was recorded by tick categories (Table 1) in order to determine the efficacy of the combination of fipronil and permethrin at preventing the establishment of tick infestation and evaluate their engorgement status. On day 6 post-infestation, the ticks were counted, removed and categorized. Following the final day 6 tick count assessments, the cage was cleaned and sprayed with an acaricide to rid the environment of any possible persistent tick infestations.

**Table 1:** **Categorization of ticks for counting (adapted from EMEA/CVMP/005/00Final-Rev.2)** [19]

**Category**	**General findings**	**Attachment status**
1	Live	Free
2	Live	Attached; unengorged*
3	Live	Attached; engorged**
4	Dead	Free
5	Dead	Attached; unengorged
6	Dead	Attached; engorged

*No filling of the alloscutum evident.

**Obvious or conspicuous filling of the alloscutum evident.

### Acaricidal efficacy

Efficacy against ticks was calculated for the administration group at each assessment day according to the formulas given below. The calculations were based on the arithmetic or geometric means of the tick counts.

Efficacy (%) = 100 × (Mc – Mt)/Mc, where:

- Mc = Arithmetic or geometric mean number of live ticks (categories 1 to 3) on dogs in the negative control group at a specific time point.
- Mt = Arithmetic or geometric mean number of live ticks (categories 1 to 3) in the treatment group at a specific time point.

The groups were compared using an ANOVA with a treatment effect after a logarithmic transformation on the tick (count + 1) data.

### Methods for *Babesia canis* detection

#### Blood smear

In case of clinical suspicion (body temperature > 39.4°C), two blood smears were prepared from a small drop of peripheral blood collected from the tip of the ear, tip of the tail or other suitable area by cutting or stabbing the skin using a new hypodermic needle. The blood smear was examined to search for intraerythrocytic piroplasms.

#### PCR analysis

Blood collected (whole blood >3.5 ml) for PCR analysis was collected in EDTA tubes. Prior to starting the procedure, approximately 1 mL of blood was taken from the whole blood sample and stored in a cryo tube in a −30°C freezer which served as a secondary sample for PCR analysis.

The remaining whole blood samples were transferred to the ClinVet molecular laboratory for analysis. Total genomic DNA was isolated from whole blood samples, using a commercial genomic DNA isolation kit. Polymerase chain reaction entailed the use of primers specific to a region of the *B. canis* rDNA (17). Up to 400 ng isolated DNA served as template for PCR amplification of the target region. PCR products were analyzed using agarose gel electrophoresis and documented. A PCR product of approximately 300 bp indicated the presence of the *B.canis* rDNA target region in the sample. Positive, negative, no template, as well as internal amplification controls, were included in each run.

#### IFA test

At least 3 mL of blood was collected from all dogs. Serum was recovered from the plain tubes and divided into primary and duplicate aliquots. Primary aliquots were stored at 2°C to 8°C for two days until assayed for *B.canis* antibodies, using a commercial IFA test. Duplicate aliquots were frozen at < −35°C. If primary aliquots were not analyzed within two days after blood collection, these aliquots were frozen at < −35°C until analysis.

### Methods for calculating the *B.canis* blocking effectiveness

An efficacy failure (successfully infected) was regarded as a dog in the Effitix® administration group that was tested serologically positive for *B.canis* antibodies and positive for *B.canis* by PCR analysis. The percentage of dogs in the negative control group that were infected was calculated to confirm the success of the model. The percentage blocking efficacy for the treatment groups was calculated as follows:

Efficacy (%) = 100 × (Tc − Tt)/Tc, where:

- Tc = Total number of infected dogs in the negative control group.
- Tt = Total number of infected dogs in the specific treatment groups.

The proportion of animals infected in each group was also compared using the chi-square test or Fischer’s exact test as applicable. SAS Version 9.3 TS Level 1 M2 was used for all the statistical analyses. The level of significance of the formal tests was set at 5%, all tests were two sided.

### Methods: experiment 1

The experiment 1 was designed to evaluate the preventive efficacy of one spot-on application of fipronil and permethrin combination (Effitix®, Virbac) in the transmission of *B.canis* by infected adult *D.reticulatus* from day 7 to day 28 after application.

The study was conducted on five groups of seven dogs each: one negative control, not treated (group 1), one group treated with the combination on day −28 (group 2), one group treated with the combination on day −21 (group 3), one group treated with the combination on day −14 (group 4), one group treated with the combination on day −7 (group 5). Animals were then challenged with a laboratory-bred *B.canis* infected *D.reticulatus* tick strain on day 0.

All the animals were observed daily from day −35 to 28 for general health conditions and clinical signs of adverse events to treatment. The dogs were observed hourly for 4 hours post-treatment for possible adverse events. The study animals were subjected to a clinical examination on day −35 and 7, 14, 21 and 28 days post tick challenge. Additionally clinical examinations were conducted on all dogs displaying clinical signs (elevated body temperature, anemia, haematuria and/or icterus) associated with babesiosis.

Rectal body temperatures were recorded daily from day 6 to 13. Two blood smears were prepared for dogs displaying abnormally high body temperatures (>39.4°C).

Blood was collected for potential PCR assay on the Days 14, 21, 28 and from dogs on the day of being diagnosed infected with *B.canis* based on blood smear evaluations.

Blood was collected for IFA test from all dogs on days 0 (prior to tick challenge), 14, 21 and 28 and serum was assayed for *B.canis* antibodies. *In situ* tick counts were performed on days 2, 3, 4, 5 and ticks were counted and removed on day 6.

### Methods: experiment 2

The experiment 2 was designed to evaluate the preventive efficacy of one spot-on application of fipronil and permethrin combination (Effitix®, Virbac) in the transmission of *B.canis* by infected adult *D.reticulatus* two days after application.

The study was conducted on two groups of eight dogs each: one negative control and one group of dogs treated on day-2. Animals were then challenged with a laboratory-bred *B.canis* infected *D.reticulatus* tick strain on day 0.

All the animals were observed daily from day −9 to 49 (except from days 29 to 32) for general health conditions and clinical signs of adverse events to treatment. The dogs were observed hourly for 4 hours post-treatment for possible adverse events. The study animals were subjected to a clinical examination on day −9 and 6, 13, 21 and +27 days post tick challenge. Additionally clinical examinations were conducted on all dogs displaying clinical signs (elevated body temperature, anemia, haematuria and/or icterus) associated with babesiosis.

Rectal body temperatures were recorded daily from days 7 to 28. Clinical examinations were conducted and two blood smears prepared for dogs displaying abnormally high body temperatures (>39.4°C).

Blood was collected for potential PCR assay on the days −9, 0, 13, 21, 27 and from dogs on the day of being diagnosed infected with *B.canis* based on blood smear evaluations. Blood was collected for IFA test from all dogs on days 0 (prior to tick challenges), 13, 21, 27, 35, 42 and 49 and serum was assayed for *B. canis* antibodies. *In situ* tick counts were performed on days 1, 2, 3 and ticks were counted and removed on day 6.

## Results

The novel combination of fipronil and permethrin administered as topical solution to 36 dogs was well-tolerated by all animals. No relevant health abnormalities - other than babesiosis – or local reactions which can be linked to the treatment, were detected in the treated animals during both studies.

### Results: experiment 1

#### Acaricidal efficacy against *D.reticulatus*

An arithmetic mean tick count of 16.6 (33.2% of ticks infested) was recorded for the untreated negative control group on day 2 indicating a vigorous tick challenge. Efficacy values (%) based on arithmetic and geometric mean tick counts for the treated groups are summarized in Table 2. From 48 h post-infestation up to three weeks post-treatment, the combination was fully effective (100%, based on arithmetic means) and it was highly effective (≥95.7%, based on arithmetic means) against infestations with *D.reticulatus* ticks for four weeks post-treatment (Group 2).

**Table 2:** **Experiment 1: Efficacy values (%) based on arithmetic and geometric mean tick counts for the treated groups**

**DAY**	**EFFICACIES (%)**			
	**GROUP 2 – Treated 28 days prior to tick challenge**		**GROUP 3 – Treated 21 days prior to tick challenge**	
	**Arithmetic mean**	**Geometric mean**	**Arithmetic mean**	**Geometric mean**
+2	95.7	97.0	100.0	100.0
+3	100.0	100.0	100.0	100.0
+4	100.0	100.0	100.0	100.0
+5	100.0	100.0	100.0	100.0
+6	100.0	100.0	100.0	100.0
**DAY**	**EFFICACIES (%)**			
	**GROUP 4 – Treated 14 days prior to tick challenge**		**GROUP 5 - Treated 7 days prior to tick challenge**	
	**Arithmetic mean**	**Geometric mean**	**Arithmetic mean**	**Geometric mean**
+2	100.0	100.0	100.0	100.0
+3	100.0	100.0	100.0	100.0
+4	100.0	100.0	100.0	100.0
+5	100.0	100.0	100.0	100.0
+6	100.0	100.0	100.0	100.0

#### Anti-feeding efficacy against *D.reticulatus*

**Figure 1:**
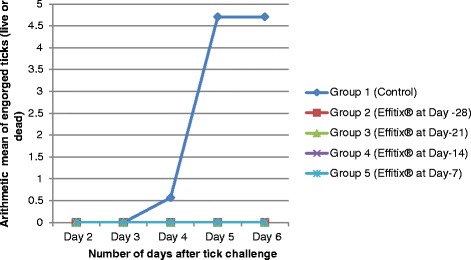
**Experiment 1: Number of engorged ticks (live or dead) for treated and control groups.** Group 1: untreated dogs; Group 2: treated on day −28; Group 3: treated on day −21; Group 4: treated on day −14; Group 5: treated on day −7. All groups were infested with ticks on Day 0.

The engorgement status of the ticks was evaluated at each tick count. No engorged tick (dead or alive) was found on the dogs treated with the combination whereas a mean of 4.7 ticks (arithmetic mean) was found on the control group. The numbers of engorged ticks per group are shown in Figure 1.

### *B.canis* blocking effect

All dogs included in the experiment were tested negative for *B.canis* antibodies in the IFA assay prior to tick infestation.The infection rate of the ticks used for infestation and confirmed by PCR analysis was high with 28% of the ticks harbouring *B.canis*. All untreated control dogs developed specific antibodies to *B.canis* and were positive for *B.canis* by PCR analysis on one or more post-challenge timepoints (Table 3). In the treated groups, only two dogs out of 28 met the criteria of a clinically significant infection (Table 3). The treatment did significantly (p < 0.05) lower the risk of infection with an average of 92.9% in preventing the transmission of *B.canis* by infected *D.reticulatus* over the challenge period (Table 4).

**Table 3:** **Summary of positive infection with** ***Babesia canis*** **based on PCR and IFA tests results**

Group*	**Animal ID**	**PCR**	**IFA**	Blood smear**	Infected***
**Results for experiment 1**					
**Group 1 Untreated**	1-1-1	**POS**	**POS**	**POS**	**YES**
	1-1-2	**POS**	**POS**	**POS**	**YES**
	1-1-3	**POS**	**POS**	**POS**	**YES**
	1-1-4	**POS**	**POS**	**POS**	**YES**
	1-1-5	**POS**	**POS**	**POS**	**YES**
	1-1-6	**POS**	**POS**	-	**YES**
	1-1-7	**POS**	**POS**	**POS**	**YES**
**Infected = 100%**					
**Group 2 Treated on day −28**	1-2-1	NEG	NEG	-	NO
	1-2-2	**POS**	**POS**	**POS**	**YES**
	1-2-3	NEG	NEG	-	NO
	1-2-4	NEG	NEG	-	NO
	1-2-5	NEG	NEG	-	NO
	1-2-6	NEG	NEG	-	NO
	1-2-7	**POS**	NEG	-	NO
**Infected = 14.3%**					
**Group 3 Treated on day −21**	1-3-1	NEG	NEG	-	NO
	1-3-2	NEG	NEG	-	NO
	1-3-3	NEG	NEG	-	NO
	1-3-4	**POS**	NEG	-	NO
	1-3-5	NEG	NEG	-	NO
	1-3-6	NEG	NEG	-	NO
	1-3-7	NEG	NEG	-	NO
**Infected = 0%**					
**Group 4 Treated on day −14**	1-4-1	NEG	NEG	-	NO
	1-4-2	NEG	NEG	NEG	NO
	1-4-3	NEG	NEG	-	NO
	1-4-4	NEG	NEG	-	NO
	1-4-5	NEG	NEG	-	NO
	1-4-6	NEG	NEG	NEG	NO
	1-4-7	NEG	NEG	-	NO
**Infected = 0%**					
**Group 5 Treated on day −7**	1-5-1	NEG	NEG	-	NO
	1-5-2	NEG	NEG	-	NO
	1-5-3	NEG	NEG	-	NO
	1-5-4	NEG	NEG	-	NO
	1-5-5	**POS**	NEG	-	NO
	1-5-6	**POS**	**POS**	**POS**	**YES**
	1-5-7	NEG	NEG	-	NO
**Infected = 14.3%**					
**Results for experiment 2**					
**Group 1 Untreated**	2-1-1	**POS**	NEG	**POS**	NO
	2-1-2	**POS**	**POS**	**POS**	**YES**
	2-1-3	**POS**	NEG	**POS**	NO
	2-1-4	**POS**	**POS**	**POS**	**YES**
	2-1-5	**POS**	**POS**	**POS**	**YES**
	2-1-6	**POS**	**POS**	**POS**	**YES**
	2-1-7	**POS**	**POS**	**POS**	**YES**
	2-1-8	**POS**	**POS**	**POS**	**YES**
**Infected = 75%**					
**Group 2 Treated on day −2**	2-2-1	NEG	NEG	NEG	NO
	2-2-2	NEG	NEG	NEG	NO
	2-2-3	NEG	NEG	NEG	NO
	2-2-4	NEG	NEG	NEG	NO
	2-2-5	NEG	NEG	NEG	NO
	2-2-6	NEG	NEG	NEG	NO
	2-2-7	NEG	NEG	NEG	NO
	2-2-8	NEG	NEG	NEG	NO
**Infected = 0%**					

*Experiment 1: Group 1: untreated dogs ; Group 2: treated on day −28; Group 3: treated on day −21; Group 4: treated on day −14; Group 5: treated on day −7.

Experiment 2: Group 1: untreated dogs ; Group 2: treated on day −2.

All groups were infested with ticks on Day 0.

**Blood smear only prepared if clinical signs observed.

***Dogs declared positive if both IFA and PCR are positive.

POS: dog with a positive IFA or PCR or blood smear analysis.

NEG: dog with a negative IFA or PCR or blood smear analysis.

**Table 4:** **Babesia blocking effect: detailed results for both studies**

	**Groups (n)**	Infected*	**Prevention (%)**
**Experiment 1**	Control (7)	7	-
	All treated (28)	2	92.9
**Experiment 2**	Control (8)	6	-
	All treated (8)	0	100

*Dogs declared positive if both IFA and PCR positive.

### Results: experiment 2

#### Acaricidal efficacy against *D.reticulatus*

An arithmetic mean tick count of 25.5 (51% of ticks infested) was recorded for the untreated negative control group on day 2 indicating a vigorous tick challenge. Efficacy values (%) based on arithmetic and geometric mean tick counts for the treated groups are summarized in Table 5. The combination was rapidly effective as more than 70% of the *D.reticulatus* ticks were killed within 24 h and was fully effective as from 72 h post-infestation.

**Table 5:** **Experiment 2: efficacy values (%) based on arithmetic and geometric mean tick counts for the treated group**

**Day**	**GROUP 2 – Treated 2 days prior to tick challenge**	
	**Arithmetic mean**	**Geometric mean**
+1	71.5%	79.9%
+2	99.5%	99.7%
+3	100.0%	100.0%
+6	100.0%	100.0%

#### Anti-feeding efficacy against *D.reticulatus*

**Figure 2:**
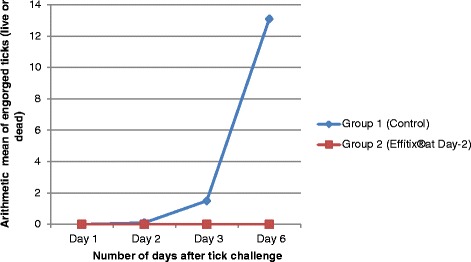
**Experiment 2: Number of engorged ticks (live or dead) for treated and control groups.** Group 1: untreated dogs; Group 2: treated on day −2. All groups were infested with ticks on Day 0.

The engorgement status of the ticks was evaluated at each tick count. No engorged tick (dead or alive) was found on the dogs treated with the combination whereas a mean of 13.1 ticks (arithmetic mean) was found on the control group. The numbers of engorged ticks per group are shown in Figure 2.

### *B.canis* blocking effect

The infection rate of the ticks used for infestation and confirmed by PCR analysis was high with 12% of the ticks harbouring *B.canis*. All dogs included in the study were tested negative for *B.canis* antibodies in the IFA assay prior to tick infestation. Six dogs out of eight untreated control dogs developed specific antibodies to *B.canis* and were positive for *B.canis* by PCR analysis on one or more post-challenge time points (Table 3). In the treated groups, no dog met the criteria of a clinically significant infection (Table 3). Therefore, the treatment was regarded to be fully effective in preventing the transmission of *B.canis* infected *D.reticulatus* ticks (100% preventive efficacy, p = 0.007, see Table 4) over this assessment period.

## Discussion

The principal findings of these studies are that a fixed combination of permethrin 54.5% and fipronil 6.1% (Effitix®) was highly effective and well tolerated for the prevention of *B.canis* transmission by infected *D.reticulatus* when administered once monthly to dogs. In addition, tick mortality was high, with a high percentage of ticks killed in less than 24 hours and with 100% of all infested ticks being dead within 3 days post challenge.

### Study design

The study design selected presented a very severe challenge scenario with a vigorous tick infestation for both studies and ticks presenting high *B.cani*s infection rates. In particular, in experiment 1 the tick infection rate was 28% (12% in experiment 2). In the field, the infection rates of *D.reticulatus* ticks should be considerably less. Recent surveys conducted in central Europe, where babesiosis is well represented, showed a prevalence of *B.canis* infections in ticks ranging from 2.3% to 14.7% in Slovakia [2] and 2.5% in the southwest of Germany [20]*.* Another advantage of this model is that the challenge load with infected ticks can be pre-determined and standardized which is not the case when dogs were either exposed to infected ticks under field conditions or when infected ticks were collected from the field and tested on dogs under controlled laboratory conditions [3].

The study design selected detected exposure to *Babesia* infection in dogs using four different diagnostic techniques: (a) regular monitoring of body temperature and health status of all dogs; (b) thin blood smear examination for *B.canis* parasites in red blood cells of pyrexic dogs (>39.4°C); (c) regular molecular assays for detection of *B. canis* DNA by PCR; (d) regular serology for *B. canis* antibodies. All diagnostic tests have inherent advantages and limitations. Regular monitoring of body temperature and health status of the dogs corresponds to the clinical course of natural infection [1]. Blood smear examination is based on current field-practice leading to a non-equivocal diagnostic as the piroplasms are identified. However, the number of intravascular organisms fluctuates over time following transmission leading to false negative results for an infected patient. Serology relies on an immunologically appropriate and detectable host immune response against one or more pathogens, one potential limitation being diminution of specificity due to antibody cross-reactivity and leading to a false positive result. PCR has the advantage to specifically target a pathogen at the species or strain level [21].

In the two studies, an efficacy failure (dogs effectively infected) was therefore regarded as a dog in the Effitix® administration group that was tested positive to display PCR, as well as IFA positive tests results. Sero-conversion is influenced by the general immune status of the host. However, since the dogs enrolled in the study were clinically healthy, immunosuppression leading to an impaired antibody response appeared unlikely. In some cases, the transient minute quantity of *B.canis* transmitted by infected ticks was detected in dogs by sensitive PCR methodology; however, this was not sufficient to lead to any further clinical sequelae and development of specific antibodies. Hence, these dogs were not considered clinically infected. As a result, a combination of both assessment criteria for clinical infection with *B.canis* was used: dogs needed to display PCR as well as IFA positive tests results. This, ensured a more meaningful evaluation of the transmission blocking effectiveness of the combination.

When compared to the first experiment, the follow-up period of the animals in the second experiment was extended up to day 49 post-tick challenge. This change in the study design was decided in order to adopt a more conservative approach in the evaluation of the efficacy of Effitix®. A late stage sero-conversion by days 28–49 may indicate a low level transmission of *B.canis* sporozoites during a brief attachment by a small number of ticks without fever or other signs of babesiosis being evident [15]. In that specific case, even if the treatment did not completely block the transmission of *B.canis* in a small proportion of dogs, it prevents the development of clinical signs of babesiosis. Therefore it can be expected that the efficacy of Effitix® in preventing the clinical signs of *B.canis* in dogs was well evaluated, based on a reliable, reproducible and highly challenging model and that the protection rate may be even higher under field conditions where prevalence of *B.canis* infected ticks is lower.

### *Acaricidal* effect

The combination provided a complete killing of *D.reticulatus* 72 h post-infestation during one month post-application. In addition, a rapid killing was documented in experiment 2, as more than 70% of the *D.reticulatus* ticks were killed within 24 hours post-infestation. A complete killing of *D. reticulatus* was already observed with the fipronil mono-product spot-on up to six weeks post-treatment [22] whereas a rapid killing against *D.reticulatus* was described with permethrin-based mono-product spot on formulations [23]. Therefore, it can be expected that the excellent and persistent acaricidal effect against *D.reticulatus* observed in both studies is linked to the persistent and robust acaricidal properties of fipronil together with the rapid killing effect of permethrin.

### *B.canis* blocking effect

The blocking effect (92.9% for experiment 1 and 100% for experiment 2) observed in these studies was similar to what was recently reported in studies using a laboratory transmission blocking model. For example, one oral anti-flea and tick product containing afoxolaner was tested on dogs. One dog out of eight was found slightly positive in the IFA test at day 93 post-treatment whereas the other dogs were IFA, blood smear and PCR negative [17]. Another study involving the combination of fipronil-amitraz-(S)-methoprene demonstrated 86% efficacy against transmission using a similar protocol [15]. Another recent study involving an imidacloprid/flumethrin collar reported a 100% protection level based on *B. canis* antibodies detection in eight treated dogs. Similarly, a 100% blocking effect over a six-months field trial was described with 9% amitraz-impregnated tick collars against *B.canis rossi* [24]. It should be noted however that one of the strength of the data reported in this article is the severity of the parasitical pressure in the laboratory model selected compared to field situation, with high tick retention rates combined with important *B.canis* infestation levels. Another strength is the number of dogs involved leading to a total of 36 dogs in treated groups and 15 dogs in the control groups taking into account the probable host variability in tick retention rate of *D.reticulatus* and *B.canis* susceptibility.

The complete and rapid killing of ticks by the tested combination (acaricidal effect) is of importance with respect to the ability to block transmission of pathogens. With 100% of all infested ticks being dead within 3 days post challenge, the combination successfully prevented transmission of mature *Babesia* sporozoites into the treated dogs, whereas they were readily transmitted to the untreated control group. Furthermore, no engorged tick (dead or alive) was found for all treated dogs during the overall assessment period whereas a mean of 4.7 ticks (arithmetic mean) was found on the control group in experiment 1 and 13.1 ticks (arithmetic mean) in experiment 2. This anti-feeding effect is certainly another important element in preventing the transmission of pathogens. When compared to the published data on the capacity of fipronil and permethrin to provide an anti-feeding effect, it should be noted that fipronil has poor anti-feeding properties [13] as dead engorged and dead attached ticks are found 48 hours post-treatment when using fipronil spot-on alone [22]. On the contrary, permethrin is well-known for its repellent effect. Permethrin has been shown to be a contact repellent meaning that ectoparasites must come in contact with the molecule to be affected. It is probable that only a few minutes of exposure are sufficient to cause the ticks to move away from treated surfaces while many of them have received a lethal dose during this period [13,14].

Therefore, it can be expected that the excellent blocking effects observed in both studies is not only due to a complete and rapid killing of the parasites but also to repellent properties linked to the presence of permethrin in the combination.

## Conclusions

In conclusion, the novel combination of fipronil and permethrin was highly effective against challenge with *D.reticulatus* ticks up to 1 month after application. The high acaricidal efficacy of the combination combined with anti-feeding properties resulted in a transmission protection level ranging from 92.9 % (experiment 1) to 100% (experiment 2) against the clinical signs of *B.canis* in dogs. This combination can be used as a part of the strategy to control flea and tick infestations, in particular when there is a risk of transmission of vector-borne diseases.
